# Effect of graphene oxide inclusion on the optical reflection of a silica photonic crystal film†

**DOI:** 10.1039/c8ra02235f

**Published:** 2018-05-04

**Authors:** Cheng Hao Lee, Jiali Yu, Yanming Wang, Alan Yiu Lun Tang, Chi Wai Kan, John H. Xin

**Affiliations:** Department of Applied Biology and Chemical Technology, The Hong Kong Polytechnic University Hung Hom Kowloon Hong Kong SAR China; Institute of Textile and Clothing, The Hong Kong Polytechnic University Hung Hom Kowloon Hong Kong SAR China kan.chi.wai@polyu.edu.hk

## Abstract

In this study, the inclusion of graphene oxide in silica photonic crystals was found to affect optical reflectance intensity and reflectance peak broadening. The quantitative relationship between weight percentage and the reflected light intensity and corresponding wavelength shift of light GO-decorated photonic crystals was studied, providing a useful parameter in the rational design of antireflection coatings for GO-based photonic crystal films. Comparison of the experimental results with a pure SiO_2_ particle film shows that a SiO_2_ particle surface layer incorporated with a fixed graphene oxide weight percentage results in broadening of the peak and a decrease in reflectance intensity. The percentage of the reduction in reflectance intensity is a function of particle size, as indicated by the structured color film surface, demonstrating the possibility of estimating the effect of different graphene oxide inclusion percentages on the antireflection properties of photonic crystal films.

## Abstract 2

In this study, the inclusion of graphene oxide in silica photonic crystals was found to affect optical reflectance intensity and reflectance peak broadening.

## Graphical abstract

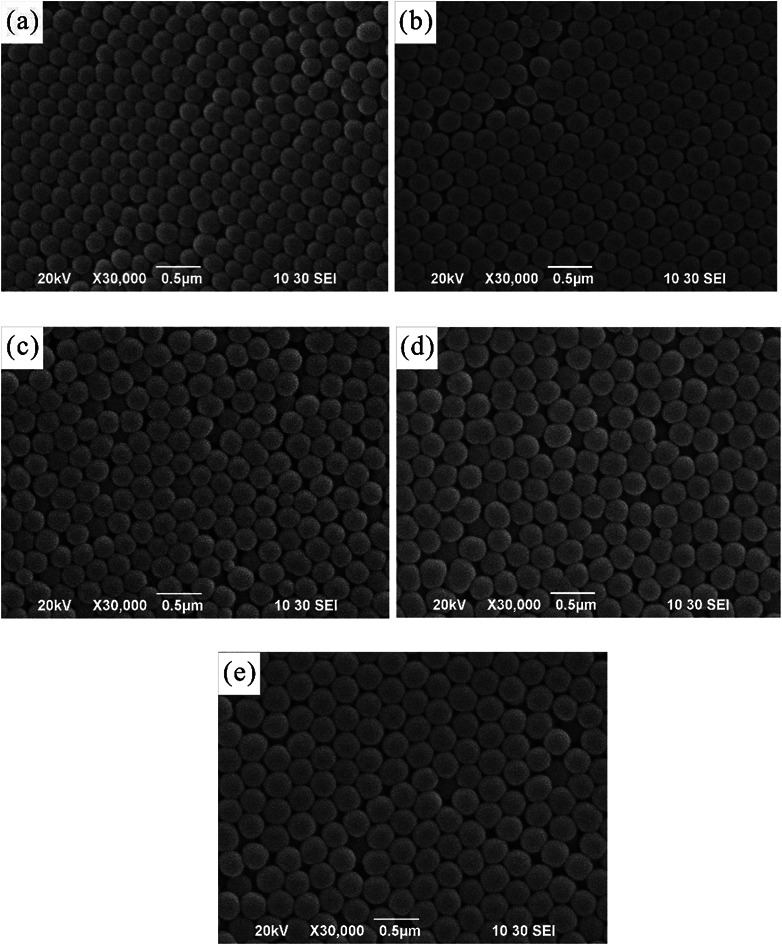

## Introduction

Photonic crystals in colloidal systems are arranged in periodic structures. These periodic structures are commonly accompanied by color reflection, depending on the periodicity.^1,2^ The colors generated from periodic physical structures are important, not only in nature but also for understanding the optical band gaps of photonic crystals.^3–6^

Visible light of specific wavelengths can be prevented from propagating in the photonic crystal structure by controlling the photonic band gap in the visible light range of 380 to 780 nm. Selective light with a specific wavelength reflected *via* the interaction between physical structure arrays and incident light is defined as structural color. Structural color reflection can be formed by dispersion, scattering, interference and Bragg diffraction,^7–10^ without any chemical colorants or pigments.

Recently, black materials have been used as additives for fabricating photonic crystals with interesting optical properties.^11,12^ General photonic crystals made from polystyrene (PS), polymethylmethacrylate (PMMA) or silicon dioxide (SiO_2_) nanoparticles used as building blocks exhibit iridescent structural colors with limited potential applications. Black materials like carbon-based materials provide a scattered light-absorbing background that can contribute to the increased coloration and antireflection properties of the material. In the case of opal-type structures, it has recently been shown that the incorporation of a light absorbing agent can enhance the structural coloration of these materials.^13–18^

The published literature provides evidence that carbon-based nanomaterials can be used as effective scattered light absorbers in a photonic crystal matrix. The inclusion of this absorber species prevents the scattering of stray light and limits the reflected light to coherent light generated by the stop band alone.^19^

Pursiainen *et al.* reported that sub-50 nm carbon nanoparticles uniformly incorporated in the interstices of highly ordered polymeric colloidal crystal films enhanced the color strength of these elastomeric films.^20^ Wang *et al.* observed an enhancement in interference colors after applying a thin carbon layer onto an anodic aluminum oxide film with well aligned nanochannels.^21^ Aguirre *et al.* observed that white opalescent poly(methyl methacrylate) colloidal crystals became uniformly and strongly colored after the incorporation of carbon black nanoparticles which acted as absorbers within the structure.^15^ The color reflection could be tuned by changing the size of the colloidal spheres, and its intensity depended on the carbon dopant loading, up to a controlled weight percentage of carbon, above which the materials became more optically absorptive.

Among the carbon-based dopants used for the improvement of optical properties, an important derivative of graphene, graphene oxide (GO), has become one of the most studied nanomaterials over the past decade, due to its stability under ambient conditions^22^ and controllable reflection or transmittance in the wide electromagnetic spectrum, ranging from UV to the near IR region.^23^ Compared with graphene, GO has many unique advantages. First of all, the optical properties of GO can be tuned dynamically by manipulating the content of oxygen-containing groups, through either chemical or physical reduction methods.^24,25^ Secondly, GO can be chemically synthesized and is solution processable. This is beneficial for low cost and scalable integration methods, such as spin coating and spraying methods, applicable for the proposed antireflection or light harvesting layer formation. Therefore, it is expected that a high performance, simple and low cost antireflection coating with selected colors can be developed using a self-assembled SiO_2_ particle film with incorporated GO.

Li *et al.* reported that GO-based sheets in an aqueous solution can form photonic liquid crystals, which display tunable colors at various GO concentrations.^26^ In addition, Eda and co-workers observed that ultrathin films of reduced GO (thicknesses ranging from a single monolayer to several layers, *i.e.* around 1–5 nm) appeared almost transparent.^27^ Structural coloration, especially color reflection in a GO dispersion, is quite unusual, considering that graphene oxide is highly polydispersed, irregularly shaped, curved in solution^28,29^ and two orders of magnitude thinner than photonic band-gap materials. The basic color reflection mechanism of GO-doped photonic crystals at a submicron scale, however, is not well understood. From a practical point of view, silica-based photonic crystals potentially allow the use of graphene oxide in various optical applications, as long as an easy method is provided for the manipulation of optical reflection.^30^ Inspired by these interesting observations, we speculated about which factors might cause a change in color intensity with the adjustment of the percentage of graphene oxide and any significant change in color reflection intensity upon the incorporation of GO into the photonic crystal array. The effect of GO inclusion on the antireflection properties of photonic crystals is still in the theoretical stage and the fabrication of graphene oxide-based photonic crystals with tunable optical reflection and reflected wavelength shift properties remains of interest for further investigation.

The optical reflectance results of GO-doped silica photonic crystal films with various particle sizes have confirmed that the amount and distribution of graphene oxide as a scattered light absorber on a SiO_2_ photonic crystal film reduces the color reflection intensity and reflected peak broadening in terms of the Full Width at Half Maximum (FWHM) value as well as the shift in reflection wavelength, rather than the grain size effect. Due to a lower extinction coefficient value compared to other carbon-based nanomaterials, the reflectance intensity in terms of the percentage of graphene oxide inclusion can be adjusted and is applicable in a range of particle sizes.

## Experimental

### Materials

The chemical reagents used in these experiments are: tetraethyl orthosilicate (TEOS) (99.0%) purchased from Sigma-Aldrich Co., LLC; ammonia (NH_3_, 25% in H_2_O) and ethanol (EtOH, 99.9%) from Fisher Scientific Co., Ltd., UK; graphene oxide (4 mg mL^−1^ dispersion in H_2_O) purchased from Sigma-Aldrich Co., LLC; distilled water (H_2_O, distilled by a USF-ELGA water purifier), which was dispensed from the laboratory facility. All of the materials were used as received without any further purification.

### Preparation of the silica photonic crystal film

Uniform silica nanoparticles (SNPs) with diameters ranging from 207 nm to 350 nm were synthesized based on the Stöber method.^31–35^ The study on the effects of TEOS, ammonia water and water on the synthesized particle sizes was described in the ESI.†

### Preparation of the silica photonic crystal film with graphene oxide inclusion

**Scheme 1:**
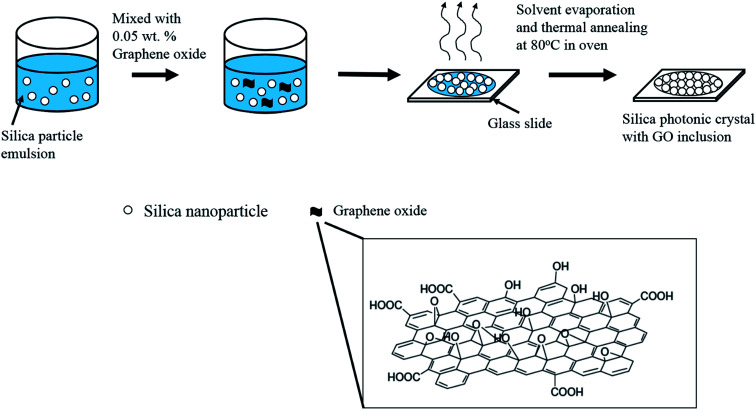
Fabrication procedure of the ordered silica particle films with graphene oxide inclusion. Graphene oxide (GO) contains a mixture of epoxy, hydroxyl, carbonyl and carboxyl groups on a carbon plane framework.

The stock solution of graphene oxide (GO) used in the preparation of the silica photonic films mixed with various weight percentages was obtained as follows: 0.3127 mL of GO solution (4 mg mL^−1^ as purchased) was dispersed in 2.5 mL of distilled water, to obtain a 0.04447 wt% GO dispersion. For the preparation of various weight percentages of GO in a silica emulsion, 53.94 μL, 89.9 μL, 125.86 μL and 153.5 μL of freshly prepared GO stock solution were mixed with 666.7 μL of 12 wt% silica emulsion. Additional quantities of distilled water were added into the mixtures containing GO and the silica emulsion to reach a final volume of 1 mL of graphene oxide containing silica solutions of 0.03%, 0.05%, 0.07% and 0.1%, respectively, and these were subsequently used to prepare the photonic crystal films. The GO–silica solution mixture was then dropped on preheated glass in an oven which was maintained at 80 °C. After 25 min, the solvent was evaporated and the GO–silica photonic crystal film was obtained (Scheme 1).

### Characterization of the silica and graphene oxide–silica photonic crystal films

Scanning electron microscopy (SEM, JSM-6490, JEOL Co. Japan) was used to observe the morphology of the silica photonic crystal films. To reduce charging effects, the samples were sputter-coated with a thin layer of gold (∼5 nm) prior to observation.

FT-Raman spectra were recorded between 1800 cm^−1^ and 300 cm^−1^ using a Bayview Raman spectrometer equipped with an optical microscope. SiO_2_ photonic crystal films, with and without 0.05 wt% graphene oxide inclusion, were irradiated with a CW green laser at 532 nm and 20 scans were captured for each sample. The spectra of silica nanoparticle films were acquired using the micro-sampling mode with a 10× objective lens and an output laser power of 150 mW. The scattered radiation was collected at 180° and the spectral resolution was set to 4 cm^−1^.

The mean particle size and polydispersity of the synthesized silica nanoparticles were measured and calculated using a particle size analyzer (Zeta Plus, Brookhaven Instruments Corp., USA). The color reflection and chromaticity coordinates (L*a*b* color chart) of the film samples were determined and analyzed using an SF 650 spectrophotometer (DataColor International, USA).

## Results and discussion

### SEM observation of the silica nanoparticle film

**Fig. 1:**
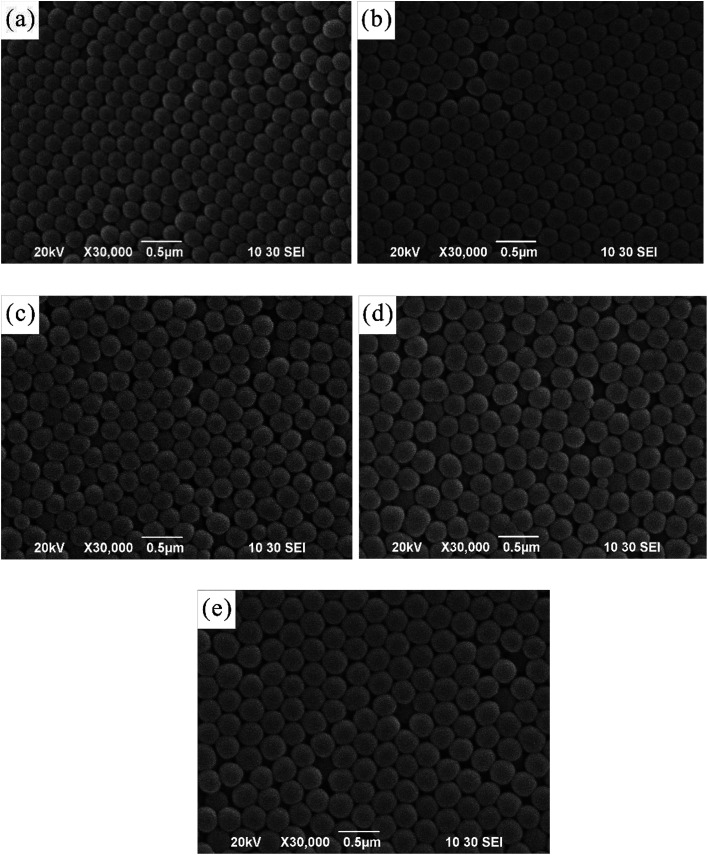
SEM micrographs of SiO_2_ nanoparticles with various particle sizes: (a) 198.8 nm; (b) 224.3 nm; (c) 232.0 nm; (d) 258.7 nm; and (e) 288.2 nm obtained using a particle size analyzer (magnification: 30 000×).

The drop-coating method enabled the silica colloids to assemble into highly periodic arrangements with a face-centered cubic (fcc) crystal driven by capillary force under slow water evaporation. It was observed that the SNPs were spherical in shape and uniform in size (the polydispersity index value was close to that of a monodispersed particle). Using the evaporation-induced self-assembly method, the arrangement of SNPs represents the face-center cubic (fcc) structure in the 〈111〉 growth direction,^1,15^ which can be seen in Fig. 1(a–e).

**Fig. 2:**
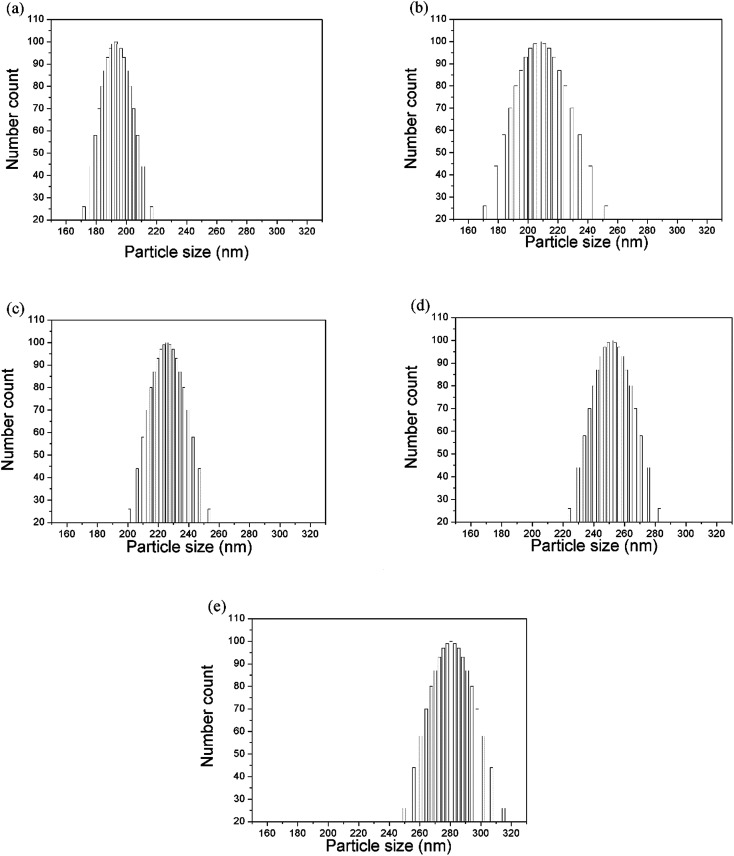
Particle size distribution (number count *vs.* particle size (diameter)) measured using a zetasizer with the mean particle sizes of (a) 198.8 nm, (b) 224.3 nm, (c) 232 nm, (d) 258.7 nm and (e) 288.2 nm.

The size (diameter) of the silica nanoparticles measured using both the particle size analyzer and the SEM micrographs is expressed in Table 1. The mean particle size was obtained after the measurement of the diameters of at least 20 particles or more from the micrographs. However, the differences in size determination using SEM and the zetasizer should, in practice, be considerably smaller because diameters measured using electron microscopy are an average between the major axis and the axis perpendicular to it, and the size data obtained using the zetasizer is orientation-averaged due to the rotational motion of the particles. However, the hydrodynamic diameter measured using the zetasizer is influenced by interactions between the particles and the dispersion media. This indicates that a few aggregates were present in the solution. The aggregates due to particle interaction observed using SEM could be attributed to the drying process during sample preparation. However, the particle size values measured using the particle size analyzer showed varied size distribution with an uncertainty of approximately 40 nm (Fig. 2).

**Table 1:** Silica particle size measured by particle size analyzer and scanning electron micrograph

Polydispersity index (PDI)	Average hydrodynamic diameter (nm) determined using the particle size analyzer	Particle diameter (nm) measured from the SEM micrograph
0.005	198.8	198.6
0.034	224.3	224.6
0.002	232.0	232.5
0.005	258.7	258.5
0.005	288.2	287.9

### Effect of optical reflectance with respect to graphene oxide weight percentages

**Fig. 3:**
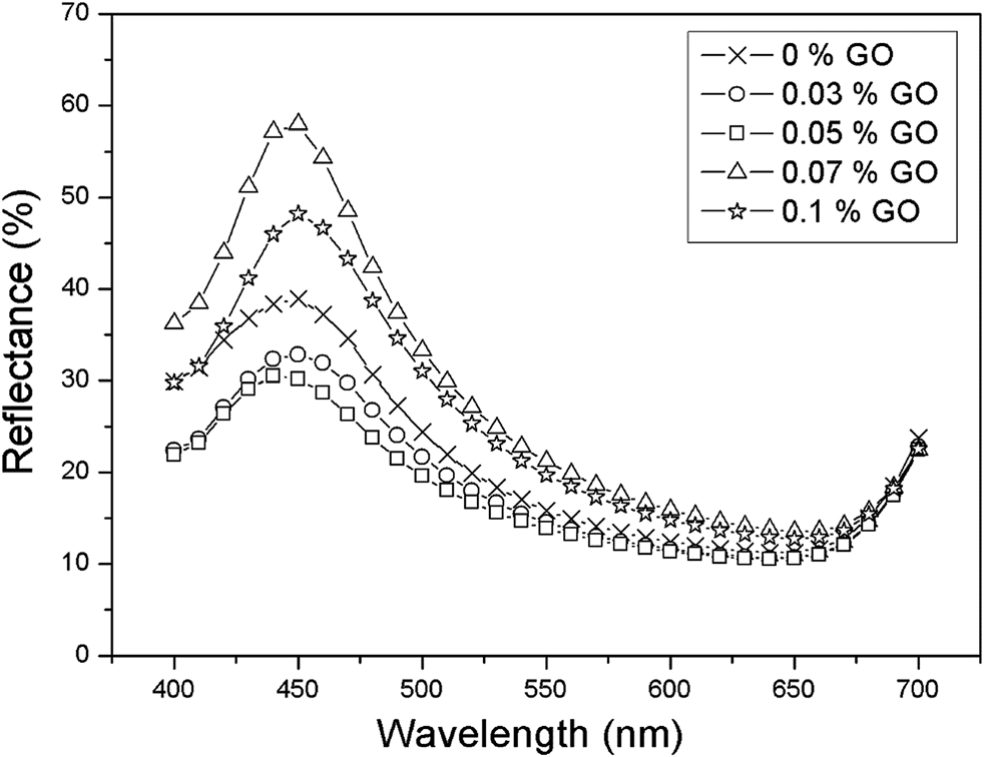
Reflectance spectrum of a pure SiO_2_ particle of 198.8 nm diameter and with various wt% graphene oxide inclusions.

We further investigated the color reflectance of graphene-oxide on a SiO_2_ layer with a mean particle size of 198.8 nm. Fig. 3 shows the reflectance of a 198.8 nm diameter particle as a function of graphene oxide percentages. However, the reflectance of the graphene oxide exhibited observable oscillations in intensity.

Oscillations of the reflectance intensity, which accompany the main Bragg peak in various dopant concentrations (Fig. 3), appear because of the interference of the light between the color reflection from the photonic crystal surface and changes in the layer periodicity of the graphene oxide sheet. We interpret these oscillations, which are observable for the aperture size of 6.6 mm in diameter, as evidence of good ordering of the measured color reflectance intensity.

The silica particle size (diameter) used for GO is 198.8 nm. The optical reflectance peak values were obtained from the weight percentage of GO, ranging from 0 wt% to 0.1 wt%. The reflectance intensity decreased when the GO dopant percentage increased, up to 0.05 wt%. This attenuation in reflectance intensity implies that the graphene oxide is involved in the absorption of scattered light at the stop band region and the arrangement of the graphene oxide sheets is more likely to be in the isotropic phase. When the GO weight percentage was above 0.05%, the color reflection intensity increased to 60%, 20% more than that of the pure SiO_2_ photonic crystal film, indicating that graphene oxide tends to be aligned from the isotropic to the nematic phase. This indicates that as the dopant weight percentage reached 0.07 wt%, the quality of the lamellar structure of graphene oxide distributed in the photonic crystal film became critical for brilliant color with maximum reflectance intensity. As the dopant weight percentage further increased to 0.1%, the reflectance intensity became lower, indicating the deterioration of the periodic lamellar structure of graphene oxide. Overall, the peak became broader in terms of the FWHM and the corresponding reflectance intensity became lower, implying that periodicity can be adjusted by the graphene oxide (GO) dopant percentage in the photonic crystal film. Consequently, the peak width and color reflection intensity decreased in the stop band close to the level of the passband due to the intensive absorption of light in the whole visible region. Fig. 3 reveals that the optimum GO weight percentage for the highest color strength (lowest color reflectance intensity) was 0.05 wt%. Apparently, the optical reflection property of the crystal film can be effectively manipulated by adding an appropriate amount of graphene oxide. This GO weight percentage, based on the result of minimal reflection (maximum white light absorption), was used as the optimal dopant amount to study the color reflection of various sizes of silica nanoparticle.

### Effects of optimal graphene oxide content on the reflectance percentages and peak profiles of photonic crystal films with various particle sizes

**Fig. 4:**
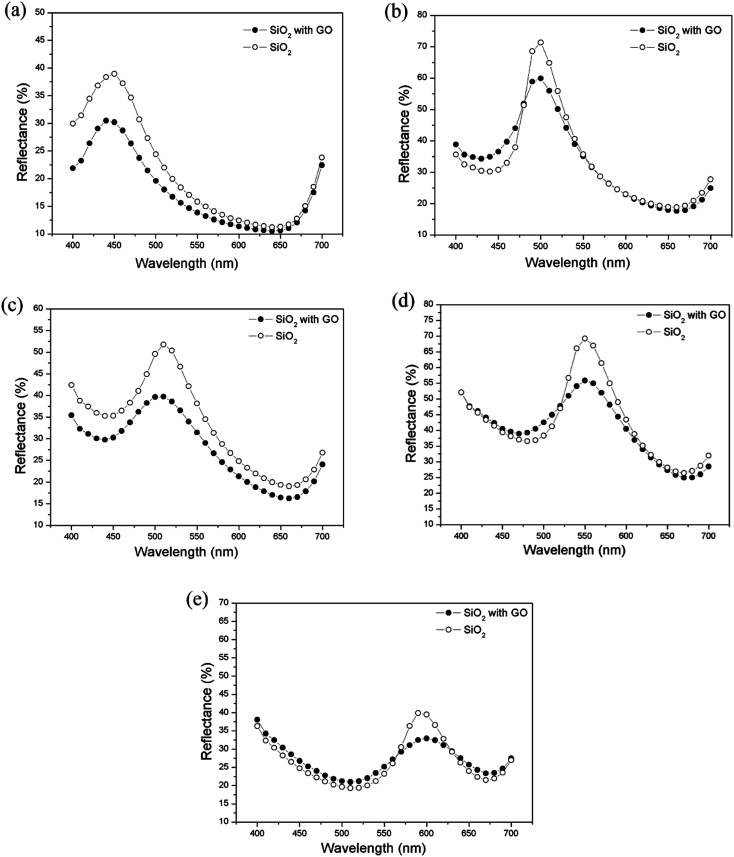
Reflectance spectra of pure SiO_2_ particles and SiO_2_ particles with 0.05 wt% graphene oxide dopant with mean particle sizes of (a) 198.8 nm, (b) 224.3 nm, (c) 232 nm, (d) 258.7 nm and (e) 288.2 nm.

To evaluate the possibility of GO functioning as an anti-reflection film, optical reflectance measurements were carried out using an SF 650 spectrophotometer (DataColor International, USA). Fig. 4 shows the reflection spectra of a silica photonic crystal film with and without graphene oxide inclusion. The changes in the effective refractive index and the filling ratio of the graphene oxide dopants affect the stop-bandwidth of the Bragg resonance in the photonic structures. The relative stop-bandwidth (Δ*λ*/*λ*_0_), where Δ*λ* is the full width at half maximum (FWHM) of the peak from the Bragg resonance at normal incidence and *λ*_0_ is the center wavelength value of the peak, can be determined. All measured reflectance peaks are baseline fitted before measuring the FWHM (Δ*λ*) and peak values (*λ*_0_). The relative stop bandwidth (Δ*λ*/*λ*_0_) for various particle sizes ranged from 4.7% to 6.7% (Table 2). The bandwidth for the particles with mean diameters of 198.8 nm and 224.3 nm matches closely with the 6.3% FWHM of the [111] crystalline plane with a relative gap width calculated by the plane wave method.^27^ For particles with diameters of 232 nm to 288.2 nm, the stop bandwidth is larger than the nominal value of the hexagonal crystalline plane (6.3%). This large deviation in relative stop bandwidth could be due to the dispersion of the colloidal silica suspension, leading to contribution from defects, cracks and disorder occurring during the self-assembly of the photonic crystal structure, thus limiting the domain size of the single-crystalline nature of the film.^27^

**Table 2:** Full Width at Half Maximum (FWHM) and reflectance peak intensity (%) of pure SiO_2_ and 0.05% graphene oxide doped SiO_2_ particles at a stopband with particle sizes ranging from 198.8 nm to 288.2 nm

SiO_2_ particle size (nm)	*λ*_max_ (nm)	FWHM value			Reflectance peak intensity (%)		
		Pure SiO_2_	SiO_2_ + 0.05% GO	Increase ↑ (%)	Pure SiO_2_	SiO_2_ + 0.05% GO	Decrease ↓ (%)
198.8	448	30.0	30.17	0.57	13.33	11.08	16.88
224.3	499.3	29.85	30.94	3.65	44.26	30.64	30.77
232.0	510.2	33.32	41.72	25.2	21.89	14.57	33.44
258.7	550	30.73	39.20	27.6	36.54	22.55	38.29
288.2	594	27.79	39.03	40.45	19.43	10.74	44.72

The reflectance spectra show that the graphene oxide doped silica photonic crystals result in a reduction in the reflectance value as a function of mean particle diameter along the 400–700 nm wavelength range. For a photonic crystal film with a larger mean particle size, more crystal defects and disorder would have been induced during self-assembly, which reduce the efficiency of the photonic stop band. Less ordered particle alignment results in a reduction of the peak reflectance value at higher percentages and a broadening of the stop band peak width. Graphene oxide in a SiO_2_ particle film with particle sizes from 198.8 nm to 288.2 nm reduces the reflectance intensity by approximately 17–45%, within the 400–700 nm range (Table 2). It is feasible that the inclusion of GO reduces the loss of light from the silica photonic crystal, resulting in better light harvesting properties. Photonic crystal films with a larger particle size, for example 288.2 nm, have different color reflection behavior compared to that with a smaller particle size, such as 198.8 nm. The volume occupied by particles of a larger size is significantly smaller than the volume occupied by smaller particles. In effect, larger particles are packed like a polydispersed sample with more voids and occupy a lower percentage of the substrate surface compared to smaller particles. However, particles with a larger size (average diameters of 258.7 nm and 288.2 nm) are less likely to be packed in a photonic crystal array with long range order. This reduction in reflectance values and broadening of the peak may be attributed to the imperfect alignment of graphene oxide distributed on the photonic crystal. The smaller particle size provided a better packing density with less disorder than that of the larger particle size, which was identified from the FWHM value of the reflected peaks (Table 2). The asymmetry in the shapes of the reflection spectra is due to domain defects in the crystalline structure of the photonic crystal. Due to light scattering at these defects, they also lead to the broadening and flattening of the spectral features.^43^ This investigation indicates the decisive role of graphene oxide in the photonic crystal structure, and thereby of the deposition scheme, in the exploitation of the color reflection characteristics of silica photonic crystals, with and without dopant.

From Table 2, it can be inferred that 0.05 wt% graphene oxide doped SiO_2_ particle films absorb even more scattered photons with respect to increasing particle size, exhibiting enhancement of their anti-reflection properties.

However, the GO layer was not very smooth and may be wrinkled, as observed using TEM. Consequently, the ARC effect of the GO could be improved upon homogeneous mixing in the SiO_2_ particle film. It is also plausible that the rough surfaces may contribute to internal reflections, which may trap light and contribute to variations in the reflectance.

It is notable that the ARC effect of GO persists over a relatively large wavelength range (from 300–1000 nm). Such a broadband response may arise due to the flake-like structure of GO mimicking a broad range of surface roughnesses with a wide range of optical parameters present in graphene oxide inclusion. However, the refractive index of GO would be expected to change with the surface roughness, which would be a function of the thickness. The influence of GO distribution on the optical response would be a promising topic of investigation, with relevance to its influence on optical limiting efficiencies for applications such as antireflection coatings.

**Fig. 5:**
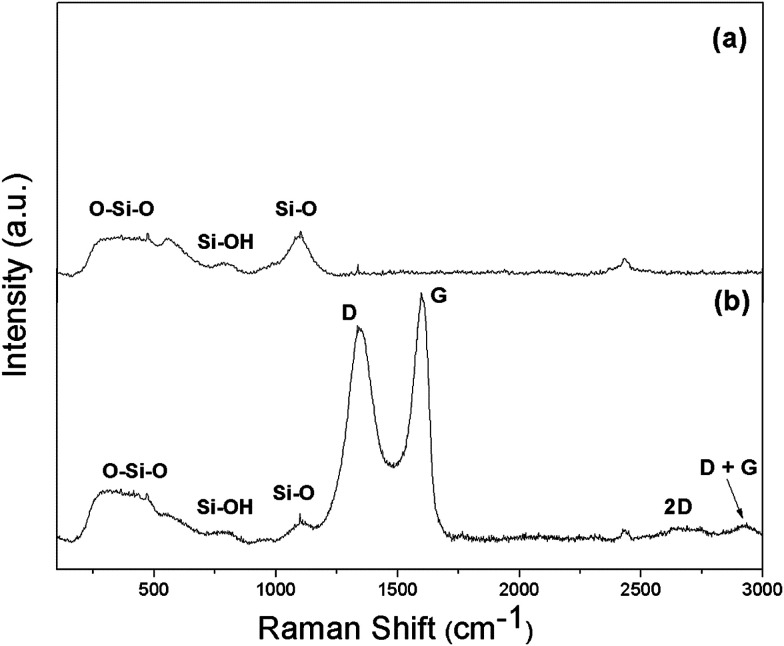
Raman spectra of (a) the synthetic silica photonic crystal film and (b) the silica photonic crystal film with 0.05 wt% graphene oxide inclusion.

Raman spectroscopy, a powerful tool, was used to identify graphene oxide inclusion in a non-destructive strategy, to examine the chemical signature of the graphene oxide in the silica photonic crystal film. As shown in Fig. 5, the most pronounced band at 470 cm^−1^ originated from oxygen atom vibrations with identical distortions of neighboring Si–O bonds,^36^*i.e.* symmetric stretching vibrations.^37^ The band at 800 cm^−1^ is also associated with the symmetric stretch vibrations of oxygen atoms, but these vibrations involve a substantial amount of surrounding Si atoms. The band at 1058 cm^−1^ is similar to those associated with the Si–O transverse and longitudinal optical modes.^37,38^ The peaks identified as the longitudinal optical mode are characteristic of the Si–O network rather than defects or impurities.

It was found that the Raman profile of the GO-decorated silica particle film exhibited sharp peaks around 1346 cm^−1^ and 1598 cm^−1^, which correspond to the well-defined D and G bands of carbon-based materials.^39^ The D band is a defect-induced Raman signature observed due to disorder or defects at the edge of graphene oxide. The G band is known to be due to sp^2^ carbon networks in the graphene oxide doped silica photonic crystal film.^40^ In addition, a 2D peak at 2700 cm^−1^ was observed.^41^

The 2D peak originates from a second-order Raman process and can be used to determine the thickness of graphene oxide layers. The intensity ratio (*I*_2D_/*I*_G_) determined from the spectrum, which is much lower than 1.9, indicates the formation of a multi-layered graphene oxide sheet structure in the sample.^42^ When 0.05 wt% of graphitic oxide is incorporated into the film, the spectrum of the silica nanoparticle film reveals not only the strong vibrational bands of the Si–O–Si network in the film but also the D and G bands of graphene oxide inclusion, indicating that the graphene oxide sheet has been distributed near the silica particle film surface.

More quantitatively, color reflection due to graphene-oxide inclusion can be evaluated by calculating the difference between the L*a*b* parameters of pure SiO_2_ and SiO_2_ doped with 0.05 wt% graphene oxide.

Table 3 summarizes the L*a*b* values of the crystal films with silica spheres of 198.8, 224.3, 232, 258.7 and 288.2 nm in average hydrodynamic diameter with 0.05 wt% of graphene oxide dopant. In order to identify the color difference after the doping of graphene oxide, the L*a*b* color space was modeled after a color-opponent theory, stating that two colors cannot be red and green at the same time or yellow and blue at the same time. As shown below, L* indicates lightness, a* is the red/green coordinate and b* is the yellow/blue coordinate.

**Table 3:** L*a*b* values of the silica crystal films, with and without graphene oxide inclusion^a^

Samples	CIE L*	CIE a*	Color shift related to CIE a*	CIE b*	Color shift related to CIE b*
198.8 nm	49.15	0.68		−29.51	
198.8 nm + GO	45.74	0.83	Red shift	−23.94	Yellow shift
224.3 nm	68.25	−36.14		2.99	
224.3 nm + GO	66.80	−28.02	Red shift	−4.43	Blue shift
232.0 nm	67.02	−23.32		−1.07	
232.0 nm + GO	61.75	−18.83	Red shift	−2.74	Blue shift
258.7 nm	77.03	−11.85		12.81	
258.7 nm + GO	74.01	−10.58	Red shift	5.94	Blue shift
288.2 nm	58.62	15.23		2.77	
288.2 nm + GO	58.31	10.95	Red shift	−0.94	Blue shift
295.3 nm	74.12	1.08		−9.32	
295.3 nm + GO	72.59	0.71	Green shift	−9.12	Blue shift

a The L* values of the crystal films do not change significantly with respect to particle size. This indicates that films of all particle sizes exhibit a darker color compared with the pure SiO_2_ particle films which have no obvious change in lightness upon the incorporation of graphene oxide. The a* and b* values indicate the chromatic change in terms of the designated color coordinates and the percentage distribution of the three primary colors.

As illustrated in Table 3, the values for the particles with average diameters from 198.8 nm to 232.0 nm increased towards the positive *x*-axis in the color chart, indicating a tendency towards red shift. However, a green shift (towards the negative *x*-axis) was observed as the average particle diameter further increased from 232.0 nm to 288.2 nm. The *b* values for the particles with average diameters from 198.8 nm to 288.2 nm decreased towards the negative *y*-axis, representing a blue shift. This blue shift of the Bragg resonance in the reflectance spectra occurs due to the decreasing of the filling ratio of the graphene oxide as particle size increased. This factor serves to increase the photonic stop-bandwidth (Δ*λ*/*λ*_0_) as the particle diameter increased, as indicated from the measured FWHM data shown in Table 2.

## Conclusions

In this study, we found that GO can improve antireflection and color strength properties and can therefore serve as a promising material for the manipulation of structural color reflection within photonic materials. By adjusting the percentage of graphene oxide inclusion and various silica particle sizes, we successfully produced GO-modified silica photonic crystals with tunable antireflection of colors, which is totally different from the traditional view that GO can only appear as transparent or dark brown. More importantly, the results demonstrate the potential applications of GO in color related fields such as light harvesting materials, textiles, paint and thin film devices with optical limiting properties.

## Conflicts of interest

There are no conflicts to declare.

## Supplementary Material

RA-008-C8RA02235F-s001
