# Supplementary material for: Effect of graphene oxide inclusion on the optical reflection of a silica photonic crystal film
Source: RSC Adv. 2018 May 4;8(30):16593–602. doi: 10.1039/c8ra02235f (PMC9080331; doi:10.1039/c8ra02235f)
Supplement: RA-008-C8RA02235F-s001 [file RA-008-C8RA02235F-s001.pdf]

Electronic Supplementary Material (ESI) for RSC Advances.
This journal is © The Royal Society of Chemistry 2018

Supporting Information

Synthesis of silica nanoparticles (SNPs)

Three types of effects have been studied in the synthesis of well dispersed silica nanoparticles. For the effect of ammonia water, a mother solution containing ethanol (130ml) and deionized water (15ml) was prepared in a 250 ml three-necked flask under magnetic stirring. TEOS (8 ml) was then added into the solution. Ammonia water (10-25 ml) was added as a catalyst 30 min later.

For the effect of water volume, a mother solution containing ethanol (130ml) and deionized water (8-25ml) was prepared in a 250 ml three-necked flask under magnetic stirring. TEOS (8 ml) was then added into the solution. 14 ml of ammonia water was added as a catalyst 30 min later.

For the effect of TEOS volume, a mother solution containing ethanol (130ml) and deionized water (15ml) was prepared in a 250 ml three-necked flask under magnetic stirring. TEOS (7-20ml) was then added into the solution. 14 ml of ammonia water was added as a catalyst 30 min later. The temperature of the mixture for all effect studies was maintained at $21^oC \pm 0.05^oC$ until the end of the reaction. All reaction mixtures were magnetically stirred with water circulation cooling until a white turbid suspension was observed.

Purification of silica nanoparticles (SNPs)

The synthesized suspension was purified by centrifugation (Centurion Scientific, K3 series) at 6000rpm for 20 min. After centrifugation, the supernatant solution was discarded and deionized water was used as dispersant for silica nanoparticles. The purification step was repeated three times to obtain pure silica nanoparticles. After purification, the purified silica nanoparticle products were re-dispersed into deionized water with 12 wt. % solid content. The

silica particle solutions were then placed in a shaking water bath for 3 hours at controlled temperature of 65°C. The silica nanoparticle solution after the aging process was used for subsequent experiments.

Table S1 Effect of ammonia water volume on silica particle sizes

Ammonia water volume (ml)	Silica particle sizes (nm)	Polydispersity Index (PDI)
10	232.4	0.013
15	227.6	0.005
20	222.2	0.005
25	198.8	0.005

where ethanol is 130ml, water is 15ml and TEOS is 8ml.

Table S2 Effect of deionized water (H_2O) volume on silica particle sizes

Deionized water volume (ml)	Silica particle sizes (nm)	Polydispersity Index (PDI)
8	161.2	0.054
10	187.0	0.005
12	189.1	0.019
15	197.9	0.005
20	190.9	0.005
25	193.6	0.008

where ethanol is 130ml, ammonia water is 14ml and TEOS is 8ml.

Table S3 Effect of TEOS volume on silica particle sizes

TEOS volume (ml)	Silica particle sizes (nm)	Polydispersity Index (PDI)
7	198.8	0.005
8	207.1	0.034
10	224.3	0.034
12	232.0	0.002
14	258.7	0.005
16	288.2	0.005
20	295.3	0.016

where ethanol is 130ml, ammonia water is 14ml and water is 15ml.
